# Association between treated/untreated traumatic dental injuries and impact on quality of life of Brazilian schoolchildren

**DOI:** 10.1186/1477-7525-8-114

**Published:** 2010-10-04

**Authors:** Cristiane B Bendo, Saul M Paiva, Cíntia S Torres, Ana C Oliveira, Daniela Goursand, Isabela A Pordeus, Miriam P Vale

**Affiliations:** 1 Department of Pediatric Dentistry and Orthodontics, Faculty of Dentistry, Federal University of Minas Gerais, Av. Antônio Carlos 6627, Belo Horizonte, MG, 31270-901, Brazil; 2 Department of Social and Preventive Dentistry, Faculty of Dentistry, Federal University of Minas Gerais, Av. Antônio Carlos 6627, Belo Horizonte, MG, 31270-901, Brazil

## Abstract

**Background:**

Traumatic dental injury (TDI) could have physical and psychosocial consequences for children. Thus, it is important to measure the impact of TDI on the quality of life of children (QoL). The aim of the present study was to investigate the association between treated/untreated TDI and the impact on the quality of life of 11-to-14-year-old Brazilian schoolchildren.

**Methods:**

A cross-sectional study was carried out involving 1612 male and female schoolchildren aged 11 to 14 years attending public and private elementary schools in the city of Belo Horizonte, Brazil. A multi-stage sampling technique was adopted to select the children. Three calibrated examiners used the Andreasen classification for the diagnosis of TDI. Oral health-related quality of life was assessed using the Brazilian version of the Child Perceptions Questionnaire (CPQ_11-14_) **- **Impact Short Form (ISF:16), composed of 16 items and self-administered by all children. Other oral conditions (dental caries and malocclusion) and the Social Vulnerability Index were determined and used as controlling variables.

**Results:**

Two hundred nineteen children were diagnosed with untreated TDI and 64 were diagnosed with treated TDI. There were no statistically significant associations between untreated or treated TDI and overall CPQ_11-14 _(Fisher = 0.368 and Fisher = 0.610, respectively). Children with an untreated TDI were 1.4-fold (95% CI = 1.1-2.1) more likely to report impact on the item "avoided smiling/laughing" than those without TDI, whereas children with a treated TDI were twofold (95% CI = 1.1-3.5) more likely to report impact on the item "other children asked questions" than those without TDI.

**Conclusions:**

Neither treated nor untreated TDI was associated with oral symptoms, functional limitations or emotional wellbeing. However, children with a TDI in the anterior teeth experienced a negative impact on social wellbeing, mainly with regard to avoiding smiling or laughing and being concerned about what other people may think or say.

## Background

The assessment of quality of life (QoL) has become an integral part of evaluating health programs. Traditional dental indicators alone (with no information on oral wellbeing) are insufficient. It is therefore important to measure the physical and psychosocial impact of oral health [1]. However, relationships between biological or clinical variables and health-related quality of life are mediated by a variety of personal, social, environmental and cultural circumstances [1,2].

Previous studies have found that traumatic dental injury (TDI) has biological, emotional and psychosocial consequences for young people [2-4]. A Brazilian case-control study found that children with fractured teeth were more likely to experience an impact on quality of life than those without injured teeth. Furthermore, children with fractured teeth were more concerned with aesthetics than function. The consequences of TDI include feeling embarrassed to smile, laugh and show teeth, difficulty in social relationships, irritability and an inability to maintain a healthy emotional state [3].

The treatment of TDI can improve the quality of life of affected children. Untreated dental injuries are more likely to have an impact on the quality of life of children than restorations, whereas crown restorations appear to contribute toward an improvement in the social aspects of QoL [4]. However, the treatment of a crown fracture does not eliminate the impact of TDI on the quality of life of children, although it may reduce it [2].

Development influences a child's comprehension regarding the relationship between health, illness and QoL, and self-awareness is age-dependent, resulting from continuous cognitive, emotional, social and language development. It is therefore fundamental to use the appropriate questionnaire to obtain information on children's oral health-related quality of life (OHRQoL) [1,5]. The first OHRQoL instrument for children between 11 and 14 years of age was the Child Perceptions Questionnaire (CPQ_11-14_) [6]. This instrument has been proven valid and reliable for use on Brazilian children [7,8].

Previous studies carried out in Brazil have investigated the impact of TDI on the QoL of children [2,3]. The two studies cited employed the Oral Impact on Daily Performances (OIDP) [9], which is not an OHRQoL measure designed specifically for children. The aim of the present study is to provide additional evidence on the association between treated/untreated TDI and its impact on the quality of life of 11-to-14-year-old Brazilian schoolchildren, using an OHRQoL instrument designed exclusively for this age group.

## Methods

### Study area and design

A cross-sectional study was carried out involving 1612 children aged 11 to 14 years attending either public or private elementary schools in the city of Belo Horizonte from September 2008 to May 2009. Participants were selected from a population of 170,289 children in the same age group enrolled at 311 public and 145 private elementary schools [10]. Belo Horizonte is the capital of the state of Minas Gerais (Brazil). It has approximately two million in habitants and is geographically divided into nine administrative districts, with considerable social, economical and cultural disparities.

The sample size was calculated to give a standard error of 2% or less, with a 95% confidence interval. To calculate the sample, a 16.1% prevalence of TDI was used [11]. A correction factor of 1.2 was used to increase the precision and a multi-stage sampling technique was adopted rather than random sampling [12]. Thus, the minimal sample size to satisfy the requirements was estimated at 1558 individuals. However, this number was increased by 20.0% (n = 1870) in order to compensate for possible refusals.

To ensure representativity, the sample was stratified according to administrative district and type of institution. The percentage distribution of 11-to-14-year-old schoolchildren in each administrative district was calculated from information provided by the local Board of Education. The distribution of participants was then determined by the proportion of this population in the respective school systems using data from samples. The first-stage was comprised of randomly selected public and private elementary schools in each administrative district of Belo Horizonte. In the second-stage, classes were randomly chosen from the selected schools.

### Measures

The research team was made up of three dentists (CBB, DG and CST), who had participated in a training and calibration exercise for each clinical condition. The Andreasen classification [13] was used to record evidence of TDI to upper and lower incisors: non-complicated fracture (enamel and enamel-dentin fracture), complicated fracture (enamel-dentin-pulp fracture), tooth dislocation (lateral luxation, intrusion and extrusion), avulsion, tooth discoloration and restoration of fractured tooth. Malocclusion and/or untreated tooth decay were identified as possible confounding variables; the diagnosis of these conditions was made using the Dental Aesthetic Index (DAI) [14] and Decayed, Missing and Filled Teeth (DMFT) Index, respectively. DMFT were visually diagnosed based on World Health Organization (WHO) recommendations [15]. Seventy-six children (not part of the study population) were randomly selected for the calibration process. Forty-four children were examined by each dentist separately for the calculation of inter-examiner agreement and 10 were re-examined after a one-month interval for the calculation of intra-examiner agreement. Kappa values ranged from 0.68 to 1.00 for inter-examiner agreement and from 0.70 to 1.00 for intra-examiner agreement, thereby demonstrating satisfactory to excellent agreement on all clinical conditions.

The testing of the methods, dental examination, administration of the questionnaires and preparation of the examiners were carried out in a pilot study involving 76 children who did not participate in the main study. The results of the pilot study indicated there was no need to change the proposed methods.

### Clinical oral examination

Dental examinations were carried out at school during daytime hours. A head lamp (Petzl Zoom head lamp, Petzl America, Clearfield, UT, USA), disposable mouth mirror (PRISMA^®^, São Paulo, SP, Brazil) and periodontal probe (WHO-621, Trinity, Campo Mourão, PA, Brazil) were used for dental examination. The dental exam was limited to visual examination and no x-rays were used. In a private room, the examiners were seated in front of the child, who remained standing. The examination for TDI included only upper and lower incisors, whereas all teeth were examined with regard to the other two oral conditions. The examiners used appropriate equipment to protect against individual cross-infection, with all necessary instruments and materials packed and sterilized in sufficient quantities for each workday.

### OHRQoL

The impact on the QoL of children was measured using the Brazilian version of the Child Perceptions Questionnaire (CPQ_11-14_) - Impact Short Form (ISF:16). The CPQ_11-14 _is part of the Child Oral Health Quality of Life (COHQoL), which is a set of questionnaires that aim to measure the impact of oral health abnormalities on the QoL of children. The CPQ_11-14 _- ISF:16 is composed of 16 items distributed among four subscales: oral symptoms, functional limitations, emotional wellbeing and social wellbeing. Each item addresses the frequency of events as applied to the teeth, lips, jaws and mouth in the previous three months. A five-point Likert scale is used, with the following options: "Never" = 0; "Once/twice" = 1; "Sometimes" = 2; "Often" = 3; and "Every day/almost every day" = 4 [6,16,17]. This instrument was adapted cross-culturally and validated for use on Brazilian children, exhibiting satisfactory psychometric properties [8]. Prior to the examination, the CPQ_11-14 _was self-administered by each child in the private room without no outside influence. The 16 items on the CPQ_11-14 _- ISF:16 questionnaire were self-administered by all children and were considered for the statistical analysis.

### Socioeconomic classification

The Social Vulnerability Index (SVI) was employed for socioeconomic classification. The SVI was developed by the city of Belo Horizonte to determine the degree of social exclusion. According to the theoretical framework that supported the development of the SVI, social vulnerability is determined based on neighborhood infrastructure and access to work, income, sanitation services, healthcare services, education, legal assistance and public transportation [18]. Thus, the SVI measures social access and determines to what extent the population of each region of the city is vulnerable to social exclusion. These scores were calculated for each district in a previous study by the city of Belo Horizonte. There are five different classes, among which Class I comprises families of the highest degree of social vulnerability (worst conditions of housing, schooling, income, jobs, legal assistance and health) and Class V is composed of families with the lowest degree of social vulnerability (best conditions). For the statistical analysis, the SVI was grouped into two categories: Classes I and II were grouped in the category "high social vulnerability" and Classes III-V were grouped in the category "low social vulnerability" [18-21].

### Ethical considerations

Following authorization from the Ethics Committee of the Federal University of Minas Gerais, permission was granted by the administration of the schools. An invitation letter was then sent to the parents of the selected children, explaining the aim, characteristics, importance and methods of the study and asking for permission for their child's participation.

### Statistics Analyses

Statistical analysis was performed using the Statistical Package for the Social Sciences (SPSS for Windows, version 15.0, SPSS Inc., Chicago, IL, USA). The impact on OHRQoL was classified as absent (CPQ_11-14 _= 0) or present (CPQ_11-14 _≥ 1), based on previous OHRQoL studies [2-4]. The Andreasen classification was used and the data were dichotomized as treated or untreated TDI for the statistical analyses. Each TDI condition was compared to children who had not suffered TDI. Oral conditions (dental caries and malocclusion) and socioeconomic classification were used as independent and controlling variables. Malocclusion was dichotomized as absent (DAI ≤ 25) or present (DAI > 25). Dental caries was dichotomized as free of teeth with untreated lesion or one or more teeth with untreated lesion. Data analysis involved descriptive statistics (frequency distribution and cross-tabulation). The chi-square test and Fisher's exact test were used to determine statistically significant associations between treated/untreated TDI and each item and the overall CPQ_11-14 _- ISF:16 score. Multiple logistic regression was used in the multivariate analysis and was performed to assess the relationship between treated/untreated TDI and the CPQ_11-14 _items, using the backward logistic model and controlling for potential confounding variables (age, gender, socioeconomic status, dental caries and malocclusion). The significance level was set at 5%.

## Results

One thousand six hundred twelve (1612) children were examined (41.7% boys and 58.3% girls), representing 11-to-14-year-old schoolchildren (53.1% 11-12 and 46.9% 13-14) in the city of Belo Horizonte, Brazil. The response rate was 86.2%. The majority of children was free of teeth with untreated lesions (72.0%), did not have malocclusion (68.7%) and lived in areas of low social vulnerability (57.8%). A total of 1337 children (82.9%) did not have any type of TDI. Two hundred eleven (13.1%) had untreated TDI alone (163 had enamel fractures, 40 had enamel-dentin fractures, five had complicated crown fractures, one had lateral luxation and two had avulsion) and 56 (3.5%) had treated TDI alone. Eight children had both treated and untreated TDI on different teeth (Table 1).

**Table 1:** Frequency distribution of the sample (n = 1612) according to variables; Belo Horizonte, Brazil, 2009

Variables	Frequency n (%)
**Gender**	
Male	672 (41.7)
Female	940 (58.3)
**Age (years)**	
11-12	856 (53.1)
13-14	756 (46.9)
**Dental caries**	
Free of teeth with untreated lesion	1161 (72.0)
One or more teeth with untreated lesion	451 (28.0)
**Malocclusion**	
Absent	1108 (68.7)
Present	504 (31.3)
**Socioeconomic status**	
Low social vulnerability	931 (57.8)
High social vulnerability	681 (42.2)
**TDI**	
Absence of fractures	1337 (82.9)
Untreated TDI	211 (13.1)
Treated TDI	56 (3.5)
Both untreated and treated TDI	8 (0.5)

To make the results easier to understand, only 10 CPQ_11-14 _items were selected for Tables 2 and 3 - pain, mouth sores, difficulty chewing, difficulty eating/drinking hot/cold foods, felt irritable/frustrated, upset, concerned with what others think, avoided smiling/laughing, teased/called names, other children asked questions. However, it is important to clarify that all 16 items were used for the statistical analysis.

**Table 2:** Frequency distribution of CPQ_11-14 _among children with untreated TDI and absence of TDI (n = 1556); Belo Horizonte, Brazil, 2009

	TDI			
			
Variables	Untreated TDI (n = 219)	Absence of TDI (n = 1337)	Unadjusted PR (95% CI)	P value
**Oral symptoms**				
Pain				
CPQ_11-14 _= 0	81 (37.0)	541 (40.5)	1	0.330^†^
CPQ_11-14 _≥ 1	138(63.0)	796(59.5)	1.1 (0.8-1.5)	
Mouth sores				
CPQ_11-14 _= 0	85 (38.8)	475 (35.5)	1	0.348^†^
CPQ_11-14 _≥ 1	134 (61.2)	862 (64.5)	0.8 (0.6-1.1)	
**Functional limitations**				
Difficulty chewing				
CPQ_11-14 _= 0	128 (58.4)	772 (57.7)	1	0.844^†^
CPQ_11-14 _≥ 1	91 (41.6)	565(42.3)	0.9(0.7-1.3)	
Difficulty eating/drinking hot/cold foods				
CPQ_11-14 _= 0	84 (38.4)	455)_34.0	1	0.212^†^
CPQ_11-14 _≥ 1	135 (61.6)	882 (66.0)	0.8 (0.6-1.1)	
**Emotional wellbeing**				
Felt irritable/frustrated				
CPQ_11-14 _= 0	138 (63.0)	827 (61.9)	1	0.743^†^
CPQ_11-14 _≥ 1	81 (37.0)	510 (38.1)	0.9 (0.7-1.2)	
Upset				
CPQ_11-14 _= 0	118 (53.9)	795 (59.5)	1	0.120^†^
CPQ_11-14 _≥ 1	101 (46.1)	542 (40.5)	1.2 (0.9-1.6)	
Concerned with what others think				
CPQ_11-14 _= 0	107 (48.9)	548 (41.0)	1	0.029^†^
CPQ_11-14 _≥ 1	112 (51.1)	789 (59.0)	0.7 (0.5-0.9)	
**Social wellbeing**				
Avoided smiling/laughing				
CPQ_11-14 _= 0	141 (64.4)	939 (70.2)	1	0.082^†^
CPQ_11-14 _≥ 1	78 (35.6)	398(29.8)	1.2(0.9-1.7)	
Teased/called names				
CPQ_11-14 _= 0	151(68.9)	913(68.3)	1	0.845^†^
CPQ_11-14 _≥ 1	68 (31.1)	424 (31.7)	0.9 (0.7-1.3)	
Other children asked questions				
CPQ_11-14 _= 0	125 (57.1)	832 (62.2)	1	0.146^†^
CPQ_11-14 _≥ 1	94 (42.9)	505 (37.8)	1.2 (0.9-1.6)	
**Overall CPQ_11-14_**				
CPQ_11-14 _= 0	5 (2.3)	19 (1.4)	0.6 (0.2-1.6)	0.368^‡^
CPQ_11-14 _≥ 1	214 (97.7)	1318 (98.6)		

^†^Chi-square test, ^‡^Fisher's exact test.

**Table 3:** Frequency distribution of CPQ_11-14 _among children with treated TDI and absence of TDI (n = 1401); Belo Horizonte, Brazil, 2009.

	TDI			
			
Variables	Treated TDI (n = 64)	Absence of TDI (n = 1337)	Unadjusted PR (95% CI)	P value
**Oral symptoms**				
Pain				
CPQ_11-14 _= 0	20 (31.2)	541 (40.5)	1	0.142^†^
CPQ_11-14 _≥ 1	44 (68.8)	796 (59.5)	1.4 (0.8-2.5)	
Mouth sores				
CPQ_11-14 _= 0	23 (35.9)	475 (35.5)	1	0.947^†^
CPQ_11-14 _≥ 1	41 (64.1)	862 (64.5)	0.9 (0.5-1.6)	
**Functional limitations**				
Difficulty chewing				
CPQ_11-14 _= 0	32 (50.0)	772 (57.7)	1	0.221^†^
CPQ_11-14 _≥ 1	32 (50.0)	565 (42.3)	1.3 (0.8-2.2)	
Difficulty eating/drinking hot/cold foods				
CPQ_11-14 _= 0	25 (39.1)	455 (34.0)	1	0.407^†^
CPQ_11-14 _≥ 1	39 (60.9)	882 (66.0)	0.8 (0.4-1.3)	
**Emotional wellbeing**				
Felt irritable/frustrated				
CPQ_11-14 _= 0	43 (67.2)	827 (61.9)	1	0.390^†^
CPQ_11-14 _≥ 1	21 (32.8)	510 (38.1)	0.7 (0.4-1.3)	
Upset				
CPQ_11-14 _= 0	41 (64.1)	795 (59.5)	1	0.464^†^
CPQ_11-14 _≥ 1	23 (35.9)	542 (40.5)	0.8 (0.4-1.3)	
Concerned with what others think				
CPQ_11-14 _= 0	28 (43.8)	548 (41.0)	1	0.661^†^
CPQ_11-14 _≥ 1	36 (56.2)	789 (59.0)	0.8 (0.5-1.4)	
**Social wellbeing**				
Avoided smiling/laughing				
CPQ_11-14 _= 0	45 (70.3)	939 (70.2)	1	0.989^†^
CPQ_11-14 _≥ 1	19 (29.7)	398 (29.8)	0.9 (0.5-1.7)	
Teased/called names				
CPQ_11-14 _= 0	48 (75.0)	913 (68.3)	1	0.258^†^
CPQ_11-14 _≥ 1	16 25.0)	424(31.7)	0.7(0.4-1.2)	
Other children asked questions				
CPQ_11-14 _= 0	31 48.4)	832(62.2)	1	0.027^†^
CPQ_11-14 _≥ 1	33 (51.6)	505 (37.8)	1.5 (1.1-2.8)	
**Overall CPQ_11-14_**				
CPQ_11-14 _= 0	1 (1.6)	19 (1.4)	1	0.610^‡^
CPQ_11-14 _≥ 1	63 (98.4)	1318 (98.6)	0.9 (0.1-6.8)	

^†^Chi-square test, ^‡^Fisher's exact test.

There were no statistically significant differences between children with untreated TDI and those without TDI regarding the overall CPQ_11-14 _- ISF:16 (Fisher = 0.368). Children with untreated TDI were 1.2-fold (95% CI = 0.9-1.6) more likely to feel "upset" and 1.2-fold (95% CI = 0.9-1.7) more likely to have "avoided smiling/laughing" than children without TDI, but these finding did not achieve statistical significance in bivariate analysis (P > 0.05). Significant differences were found between children with untreated TDI and those without TDI on the item "concerned with what others think" (P = 0.029). Children with untreated TDI reported less of an impact from this item than those without TDI (PR = 0.7, 95% CI = 0.5-0.9) (Table 2).

In the comparison of children with treated fractures and those without TDI (Table 3), there was no association to the overall CPQ_11-14 _- ISF:16 score (Fisher = 0.610). Dental pain and difficulty chewing were more prevalent among children with treated teeth than those with no TDI, but this difference did not achieve statistical significance (P > 0.05). On social wellbeing subscale, only the item "other children asked questions" was statistically associated to treated teeth (PR = 1.5, 95% CI = 1.1-2.8, P = 0.027).

In the multiple logistic regression (Table 4), separate CPQ_11-14 _- ISF:16 items and overall score were adjusted for age, gender, socioeconomic status, dental caries and malocclusion. When considering untreated TDI, the items "concerned with what others think", "avoided smiling/laughing" and "other children asked questions" remained in the model, but only two achieved statistical significance. Children with untreated TDI were 1.4-fold (95% CI = 1.1-2.1, P = 0.019) more likely to "avoided smiling/laughing" than children without TDI. However, children with untreated TDI reported less of an impact from the item "concerned with what others think" (PR = 0.6, 95% CI = 0.4-0.8, P = 0.003) than those without TDI. For treated TDI, three items continued in the model ("pain", "difficulty chewing" and "other children asked questions"). Children with a treated TDI were twofold (95% CI = 1.1-3.5) more likely to report an impact regarding the item "other children asked questions" than those without TDI; this difference was statistically significant (P = 0.012).

**Table 4:** Multiple logistic regression models explaining the influence of TDI on each item of CPQ_11-1__4_; Belo Horizonte, Brazil, 2009

Variables	Unadjusted PR (95% CI)	**Adjusted PR (95% CI)**^§^	P value
**Untreated TDI**			
Concerned with what others think			
CPQ_11-14 _= 0	1	1	0.003
CPQ_11-14 _≥ 1	0.7(0.5-0.9)	0.6 (0.4-0.8)	
Avoided smiling/laughing			
CPQ_11-14 _= 0	1	1	0.019
CPQ_11-14 _≥ 1	1.2 (0.9-1.7)	1.4 (1.1-2.1)	
Other children asked questions			
CPQ_11-14 _= 0	1	1	
CPQ_11-14 _≥ 1	1.2 (0.9-1.6)	1.2 (0.9-1.7)	0.111
**Treated TDI**			
Pain			
CPQ_11-14 _= 0	1	1	0.214
CPQ_11-14 _≥ 1	1.4 (0.8-2.5)	1.4 (0.8-2.6)	
Difficulty chewing			
CPQ_11-14 _= 0	1	1	0.172
CPQ_11-14 _≥ 1	1.3 (0.8-2.2)	1.4 (0.8-2.5)	
Other children asked questions			
CPQ_11-14 _= 0	1	1	0.012
CPQ_11-14 _≥ 1	1.6 (1.1-2.8)	2.0 (1.1-3.5)	

PR: Prevalence ratio

CI 95%: Confidence interval

^§^Adjusted for control variables (age, gender, socioeconomic status, dental caries and malocclusion)

## Discussion

The present cross-sectional survey found that overall CPQ_11-14 _- ISF:16 was not associated to TDI, which corroborates a Canadian study on 11-to-14-year-old children that employed the same questionnaire to measure OHRQoL [4]. Another study involving Canadian children found differences in the impact of TDI according to socioeconomic status. In children from higher income groups, there were no differences in CPQ_11-14 _scores for children with and without TDI. However, the differences were significant for children in the lowest income group [22]. Although the data of the present study were adjusted for socioeconomic status, there was no association between TDI and overall CPQ_11-14 _- ISF:16.

Previous studies carried out in Brazil have found a statistically significant association between OHRQoL and children with untreated [3] and treated teeth with TDI [2], adjusting for the same controlling variables (untreated dental caries, malocclusion and socioeconomic status). These studies used a different instrument to measure the impact of TDI on OHRQoL, the OIDP [9]. The age group of these two studies was similar to that of the present study, but the instrument had been designed for use on adults. Thus, the difference between the findings of the present study and these previous Brazilian studies [2,3] may be explained by the choice of OHRQoL instrument.

TDI related to biological factors alone, regardless of social patterns, may be largely ineffective information. It is likely that multifaceted disorders have numerous risk factors working together and it is important to consider possible correlations with confounding variables [23,24]. Similarly, the presence of dental caries and malocclusion could be correlated with the occurrence of TDI and these oral conditions could have an influence over an individual's QoL [2,4,22]. Thus, these variables should be considered confounding variables and included in the multiple logistic regression due to their clinical-epidemiological significance, regardless of their statistical significance.

The association between treated/untreated TDI and impact on QoL in the present study was stronger with regard to social wellbeing than oral symptoms, functional limitations, and emotional wellbeing. Similar results were found in a Canadian study, which demonstrated the children with untreated dental injuries experienced a greater social impact on daily living than those without injuries [4].

The children in the present study diagnosed with untreated TDI felt greater dissatisfaction with their appearance than those without TDI, especially with regard to smiling and laughing. Children with untreated TDI were 1.4-fold more likely to avoid smiling or laughing than children without TDI. Restorations were found not to eliminate the impact of TDI on QoL, especially with regard to social wellbeing. However, children with restored anterior teeth worried about what the other people may think and ask about their teeth, lips, jaws or mouth. In a Canadian study, the restoration of TDI was found to reduce the impact on social wellbeing [4]. This difference between studies may be explained by the aesthetic conditions of the restorations. In a developing country such as Brazil, access to public health care services is limited and it is difficult to maintain restorations, which is indispensable to aesthetics.

The present study demonstrates that the main concerns of these children involve social interactions and are related to the perception of others regarding their dental appearance. At 11 to 14 years of age, relationships between peers are important components of an individual's perceptions regarding health and quality of life. Thus, judgments on the part of peer groups can affect an individual's emotional state and relationships with others [25]. Dental-facial aesthetics plays an important role in social interaction and psychological wellbeing among adolescents [26]. Health and quality of life experienced by an individual are not determined only by the nature and severity of the disease/disorder. The social environment, relationships and pertaining to a group of friends are important factors in early adolescence. At this age, any alteration in dental aspects can have a negative impact on QoL [25,27].

In general, TDI was weakly associated to the OHRQoL of children. Although the majority of the items on the CPQ_11-14 _- ISF:16 did not have statistically significant associations with treated/untreated TDI in the bivariate and multivariate analyses, three items did achieve statistically significant associations. However, one of these items revealed that children with untreated TDI were less likely to be concerned with what others think about their teeth, lips, jaws or mouth than those without TDI. Perhaps children without TDI tend to be more careful with their teeth and suffer less TDI due to their concern for what others think. On the other hand, this result could be a sign an ineffective instrument for measuring the impact of TDI on the OHRQoL of children, as it is not a condition-specific instrument.

Since the CPQ_11-14 _- ISF:16 is a generic measure and was not specifically designed for TDI, the lack of an association between TDI and overall CPQ_11-14 _as well as the majority of individual items may reflect this limitation. Some items may not necessarily be relevant to children with TDI. Thus, it is possible that the CPQ_11-14_-ISF:16 was unable to discriminate between children with and without TDI accordingly. It may therefore be time to develop of a TDI-specific OHRQoL instrument and further studies in this direction should be encouraged.

We recognize that the impact observed on OHRQoL could be from other oral conditions. In order to minimize this bias, we controlled the data for potential confounding variables, such as untreated dental caries and malocclusion.

## Conclusions

Neither treated nor untreated TDI was associated to oral symptoms, functional limitations or emotional wellbeing. However, children with these oral conditions in the anterior teeth were more likely to experience a negative impact on social wellbeing, especially with regard to avoiding smiling or laughing and beings concerned about what other people may think or say. This information is clinically relevant and making these results public may therefore be useful.

## Abbreviations

TDI: Traumatic dental injury; QoL: Quality of life; OHRQoL: Oral health-related quality of life; CPQ_11-14_: Child Perceptions Questionnaire; ISF:16: Impact Short Form; SVI: Social Vulnerability Index; DAI: Dental Aesthetic Index; DMFT: Decayed, Missing and Filled Teeth; COHQoL: Child Oral Health Quality of Life; OIDP: Oral Impact on Daily Performances.

## Competing interests

The authors declare that they have no competing interests.

## Authors' contributions

CB, SP, CT, DG, IP and MV conceptualized the rationale and designed the study. CB, SP, CT, AO, DG and MV performed the data collection, statistical analysis and interpretation of the data. CB, SP, CT and DG conducted the literature review and drafted the manuscript. All authors read and approved the final manuscript.
